# A Highly Sensitive Resistive Pressure Sensor Based on a Carbon Nanotube-Liquid Crystal-PDMS Composite

**DOI:** 10.3390/nano8060413

**Published:** 2018-06-08

**Authors:** Jin Pan, Shiyu Liu, Yicheng Yang, Jiangang Lu

**Affiliations:** National Engineering Lab for TFT-LCD Materials and Technologies, Department of Electronic Engineering, Shanghai Jiao Tong University, Shanghai 200240, China; pjpjpj@sjtu.edu.cn (J.P.); syliu@sjtu.edu.cn (S.L.); yichengyang@sjtu.edu.cn (Y.Y.)

**Keywords:** high sensitivity, polymer-dispersed liquid crystal (PDLC), multi-walled carbon nanotube (MWCNT), resistive pressure sensor

## Abstract

Resistive pressure sensors generally employ microstructures such as pores and pyramids in the active layer or on the electrodes to reduce the Young’s modulus and improve the sensitivity. However, such pressure sensors always exhibit complex fabrication process and have difficulties in controlling the uniformity of microstructures. In this paper, we demonstrated a highly sensitive resistive pressure sensor based on a composite comprising of low-polarity liquid crystal (LPLC), multi-walled carbon nanotube (MWCNT), and polydimethylsiloxane (PDMS) elastomer. The LPLC in the PDMS forms a polymer-dispersed liquid crystal (PDLC) structure which can not only reduce the Young’s modulus but also contribute to the construction of conductive paths in the active layer. By optimizing the concentration of LC in PDMS elastomer, the resistive pressure sensor shows a high sensitivity of 5.35 kPa^−1^, fast response (<150 ms), and great durability. Fabrication process is also facile and the uniformity of the microstructures can be readily controlled. The pressure sensor offers great potential for applications in emerging wearable devices and electronic skins.

## 1. Introduction

Pressure sensors have attracted intensive attention for applications in smartphones, electronic skins, and wearable electronic devices [1,2,3]. According to different transduction mechanisms, pressure sensors are divided into resistive, capacitive and piezoelectric types, etc. Capacitive pressure sensors are typically implemented by changing the distance or overlap between two parallel plate electrodes. They usually exhibit high linearity and high transparency, but must be well shielded to reduce electromagnetic interference (EMI). Piezo-electric pressure sensors respond to applied pressure with charge accumulation in piezoelectric materials and usually have high sensitivity. However, they are only sensitive to dynamic pressures. Resistive pressure sensors which transduce applied pressure into changes in resistance are intensively investigated because of their easy read-out mechanism, simple structure and fast response speed [4,5,6,7,8,9,10]. Resistive pressure sensors based on solid composites of elastomers and conductive fillers usually show poor sensitivity due to high Young’s modulus. Recently, several new designs using microstructures, such as porous structure [3], pyramidal structure [11], inter-locking nanofibers [9], porous carbon nanotube sponges [12], gold-film-polyurethane sponges [13], and hollow spheres-based conductive polymers [14], have been proposed to improve pressure sensitivity. Introducing such microstructures into the active layer can decrease the Young’s modulus and increase the relative change in contact area, resulting in higher sensitivity. However, difficulties and complexities in fabricating and controlling the microstructures still remain.

In this study, a facile method is presented to fabricate resistive pressure sensors based on a composite consisting of multi-walled carbon nanotube, low-polarity liquid crystal, polydimethylsiloxane (MWCNT-LPLC-PDMS). The LPLC forms polymer dispersed liquid crystal (PDLC) droplets in the PDMS elastomer, which can reduce its modulus and mean-while help to construct conductive paths in the active layer. The all-solution-based method is compatible with low-cost and large-scale manufacturing. Furthermore, characteristics and uniformity of the droplets can be readily controlled.

## 2. Materials and Methods

**Figure 1:**
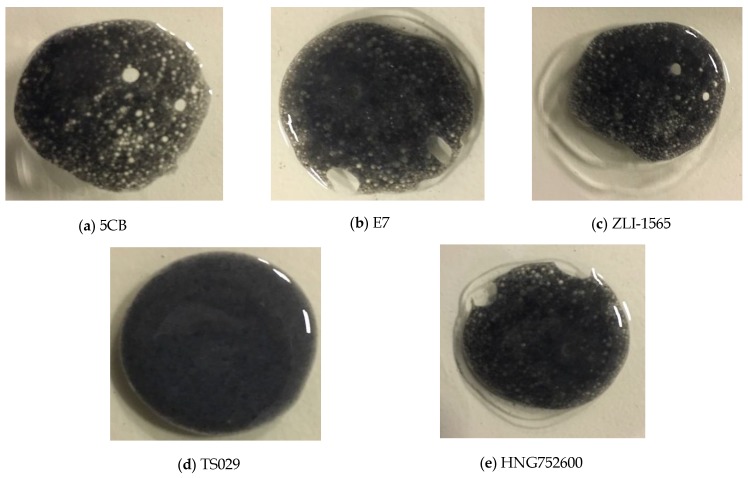
The mixture of polydimethylsiloxane (PDMS); multi-walled carbon nanotubes (MWCNTs); and (**a**) 5CB, (**b**) E7, (**c**) ZLI-1565, (**d**) TS029, and (**e**) HNG752600 after the mixture was stirred for 60 min and then left standing for 30 min.

In polymer-dispersed liquid crystal (PDLC), the LC molecules are dispersed in droplets combined with polymers. As LC droplets have a smaller elastic constant than the surrounding polymers, PDLC has an appropriate structure to get a low Young’s modulus compared with the pure polymers. The MWCNT is applied as the conductive fillers for its great conductivity and toughness. Among various dielectric elastomers for the active layer of pressure sensors, PDMS is a promising material, for its excellent elasticity and biomedical compliance with human tissues after cured. The curing ratio of PDMS was set as 30:1 to achieve a good mechanical strength. The miscibility between LC molecules and PDMS is the key issue for fabrication of the resistive sensors. Different kinds of LC molecules, including 5CB (Δ*n* = 0.154, Δε = 13.3, Merck), E7 (Δ*n* = 0.22, Δε = 14.33, HCCH), ZLI-1565 (Δ*n* = 0.1262, Δε = 7, Merck), TS029 (Δ*n* = 0.09, Δε = 0, HCCH), and HNG752600 (Δ*n* = 0.088, Δε = −8.5, HCCH) were selected to mix with PDMS and MWCNT. After the mixture was stirred for 60 min and subsequently left standing for 30 min, we found that except TS029, other four kinds of LCs cannot be well mixed with PDMS and MWCNT, as shown in Figure 1. Compare to other four kinds of LCs, the polarity of TS029 is relatively low and similar to that of PDMS because the molecules’ end-groups of TS029 mixture have extremely weak dipolar moments, as shown in Table 1. Therefore, the low-polarity TS029 has a high miscibility with PDMS which was selected to form the PDLC.

**Figure 2:**
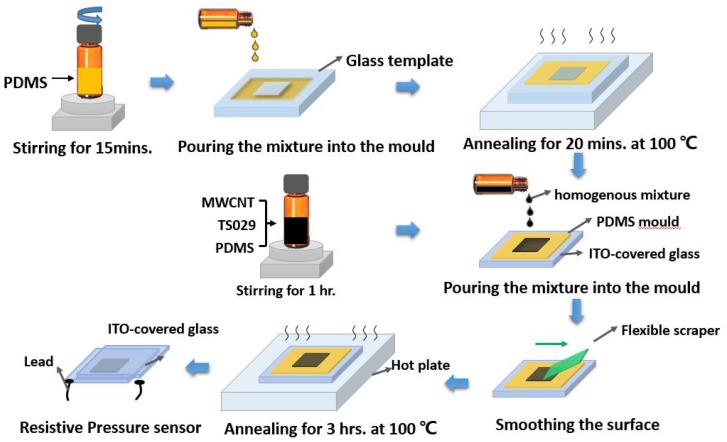
The fabrication process of the resistive pressure sensor.

Figure 2 shows the fabrication process of the resistive pressure sensor based on the MWCNT-LC-PDMS composite. To obtain an active layer with a thickness of 2 mm, we used a PDMS film with a hollowed area of 8 mm × 8 mm × 2 mm as a mould which can be easily peeled off from the glass. Initially, the mixture of PDMS (pre-polymer: curing agent = 10:1) was stirred for 15 min at room temperature and then put into a vacuum chamber for 15 min. The PDMS mould was fabricated after the PDMS mixture was annealed for 20 min at 100 °C in a glass mould. After that, the PDMS mould was coated onto an indium-tin-oxide (ITO)-covered glass with no air bubbles between two layers. After fabricating the PDMS mould, a mixture of PDMS (pre-polymer: curing agent = 30:1), MWCNT (2.5 wt % of PDMS), and TS029 was stirred for 1 h at room temperature until it became homogeneous. The concentration of TS029 in PDMS ranges from 0 to 60 wt % with a step of 5 wt %. Then the mixture was put into a vacuum chamber for 15 min to remove the air in the mixture. The MWCNT-LC-PDMS solution was drop-casted into the PDMS mould which was attached to an indium-tin-oxide (ITO)-covered glass. And the surface of mixture was smoothed by a flexible scraper, keeping height of the mixture the same as that of the PDMS mould. Then the whole mould was annealed at 100 °C for 3 h until the MWCNT-LC-PDMS composite was completely cured. Finally, the PDMS mould was peeled off and the resistive pressure sensor was fabricated by attaching a second ITO glass onto the MWCNT-LC-PDMS composite.

## 3. Results and Discussion

According to the percolation theory, we can deduce that during the mixing process, the MWCNT particles were dispersed into both PDMS and TS029 droplets, and caused TS029 to be conductive. Otherwise the resistance of the pressure sensor will increase with the concentration of TS029 since pure TS029 is non-conductive, which does not agree to our experimental results. Therefore, the conductive TS029 droplets and PDMS form a binary system which has a percolation threshold.

**Figure 3:**
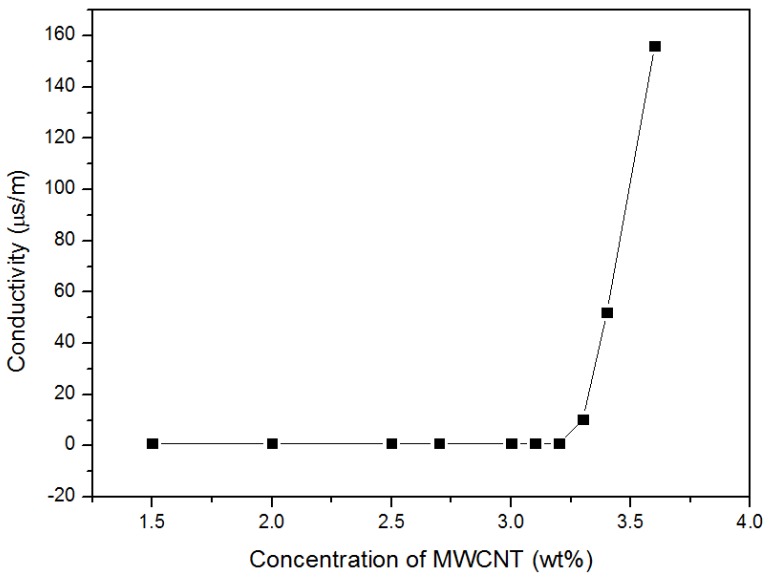
The conductivity of MWCNT/PDMS composite as a function of the concentration of MWCNT.

**Figure 4:**
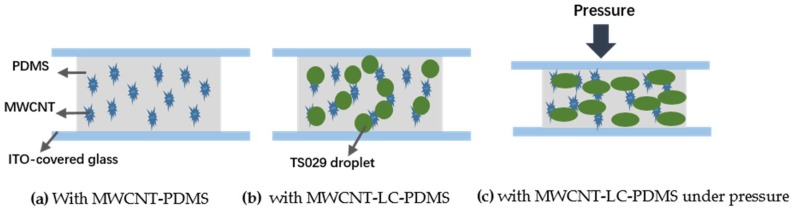
Schematic diagrams of the pressure sensor (**a**) with MWCNT-PDMS composite (**b**) with MWCNT-LC-PDMS composite (**c**) with MWCNT-LC-PDMS composite under applied pressure.

With no TS029 introduced, the MWCNTs dispersed into the PDMS can hardly form effective conductive paths and get the active layer to be conductive until the concentration of MWCNT in PDMS reached the percolation threshold, about 3.3 wt %, as shown in Figure 3. After introducing TS029 into PDMS-MWCNT composite, the MWCNT can be uniformly dispersed into the LC droplets and make the LC droplets conductive. The size of the droplets is much larger than the MWCNT particles so that the MWCNTs in PDMS can get connected more easily to build the conductive paths because the conductive droplets play a role of bridges between different MWCNT particles. When the concentration of TS029 in PDMS increased to 25 wt %, conductive paths were not effectively built up and the resistance of pressure sensors was still larger than 20 MΩ. When the concentration of TS029 increased to the percolation threshold (approximately 30 wt %), both the MWCNT particles in PDMS and the conductive TS029 droplets contributed to the construction of conductive paths, and the resistance of pressure sensors decreased significantly to less than 2 MΩ, as showed in Figure 4b. As the concentration of TS029 in PDMS further increased, more conductive paths will be built and the resistance of pressure sensors decreased.

**Figure 5:**
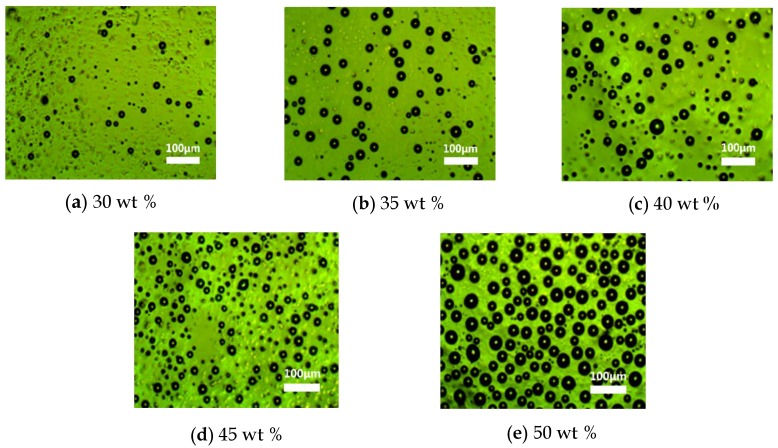
Morphology of the multi-walled carbon nanotubes-liquid crystal-polydimethylsiloxane (MWCNT-LC-PDMS) composite with concentrations of TS029 in PDMS of (**a**) 30 wt %, (**b**) 35 wt %, (**c**) 40 wt %, (**d**) 45 wt %, and (**e**) 50 wt %.

**Figure 6:**
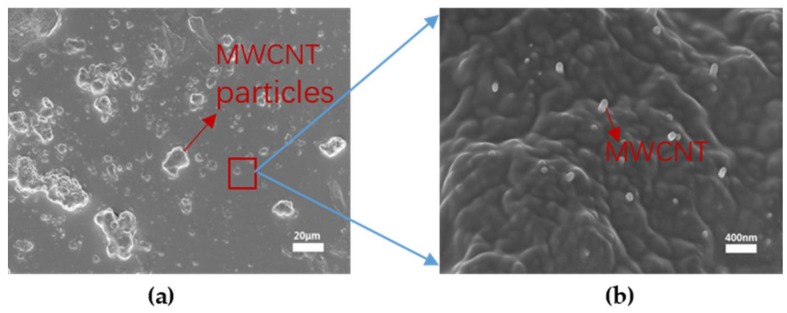
The SEM image showing the distribution of MWCNT in the composite. (**a**) the protrusions on the surface of PDMS layer formed by MWCNT particles combined with PDMS; (**b**) Several MWCNTs exposed out of the PDMS layer.

Figure 5 shows the morphology of the MWCNT-LC-PDMS composite with the concentration of TS029 in PDMS from 30 wt % to 50 wt % under a polarized optical microscope (POM, XPL-30TF, Shanghai WeiTu Optics & Electron Technology Co., Ltd., Shanghai, China). TS029 in PDMS formed many spherical droplets due to the features of PDLC. The size of these droplets was in the range of 25–32 μm which showed a good uniformity and reduced the complexity in fabricating the microstructures. Figure 6 shows the distribution of MWCNT in the composite. The MWCNT particles combined with PDMS formed some small protrusions on the surface of PDMS layer, as shown in Figure 6a. And several MWCNTs exposed out of the PDMS layer, as shown in Figure 6b. The density of LC droplets among the active layer increased with the concentration of TS029 in PDMS. When the concentration of TS029 in PDMS was higher than 50 wt %, PDMS was hard to be cured. Therefore, the concentration of TS029 in PDMS was confined in the range of 30–50 wt % for the fabricated resistive pressure sensors.

**Figure 7:**
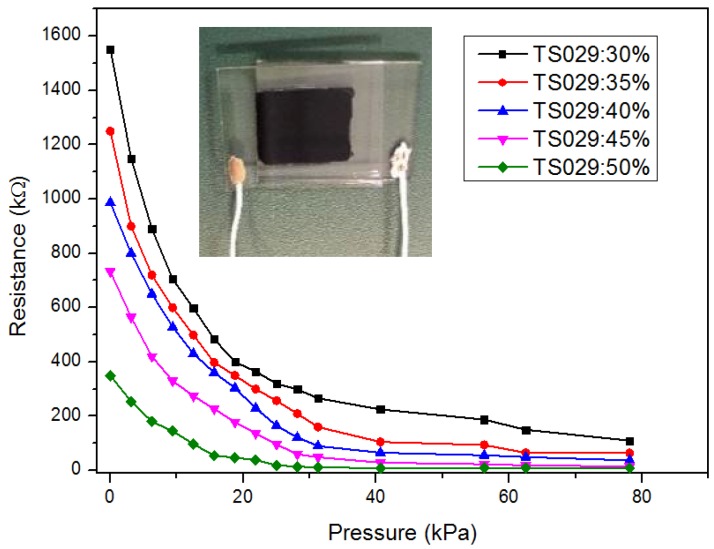
The observed resistance of the pressure sensors with different concentration of TS029 in PDMS in the pressure range of 0–80 kPa.

**Figure 8:**
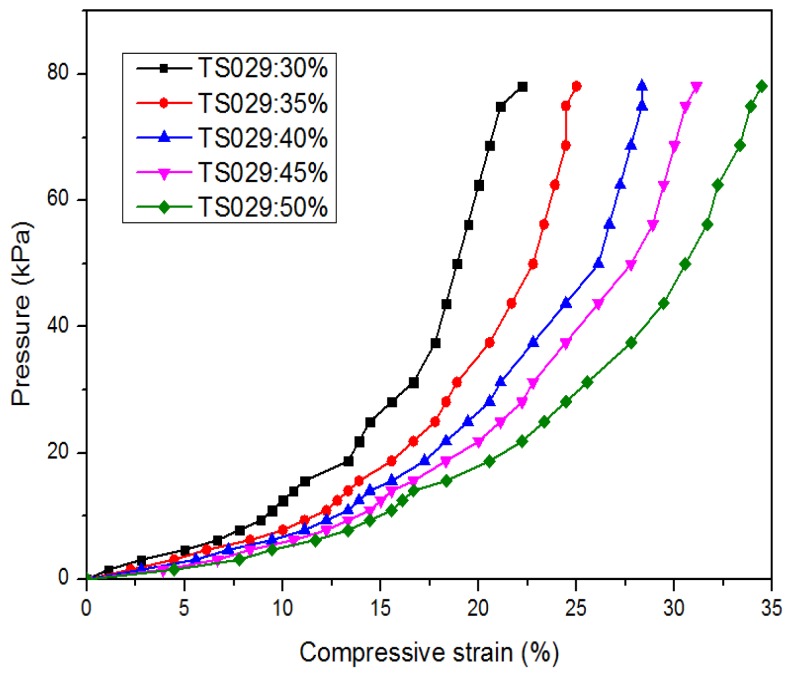
The compressive strain of the pressure sensors as a function of the applied pressure.

Figure 7 shows the performance of piezo-resistivity of the sensor in the pressure region of 0–80 kPa. The resistance of the sensor decreased as the applied pressure increased due to the reduction in conductive path length and the contact resistance between TS029 droplets and MWCNT particles (Figure 4c). With the concentration of TS029 in PDMS of 30 wt %, the sensitivity of the pressure sensor is 0.63 kPa^−1^ and 0.057 kPa^−1^ in the pressure range of 0–15 kPa and 15–80 kPa, respectively. The pressure sensitivity was defined as S = (ΔR/R_0_)/ΔP, where P denotes the applied pressure, R denotes the resistance under pressure, and R_0_ denotes the minimum resistance with applied pressure. As the concentration of TS029 in PDMS increased from 30 wt % to 50 wt %, the sensitivity of the pressure sensors increased by a factor of 8.49 times and 2.3 times in the pressure range of 0–15 kPa and 15–80 kPa, respectively. The dramatic increase in sensitivity can be attributed to the optimization in establishing the conductive path and the reduced Young’s modulus with TS029 introduced. On one hand, the increased concentration of TS029 in PDMS leads to a higher density of conductive LC droplets so that the conductive paths are much easier to be built to achieve a better sensitivity. On the other hand, because the elastic constant of the LC droplet is less than that of PDMS layer, a higher proportion of LC in composite will lead to a lower Young’s modulus. As illustrated in Figure 8, the Young’s modulus (the slope of the pressure-strain curve) deceases as the concentration of TS029 in PDMS increases, resulting in the improvement of the sensitivity.

**Figure 9:**
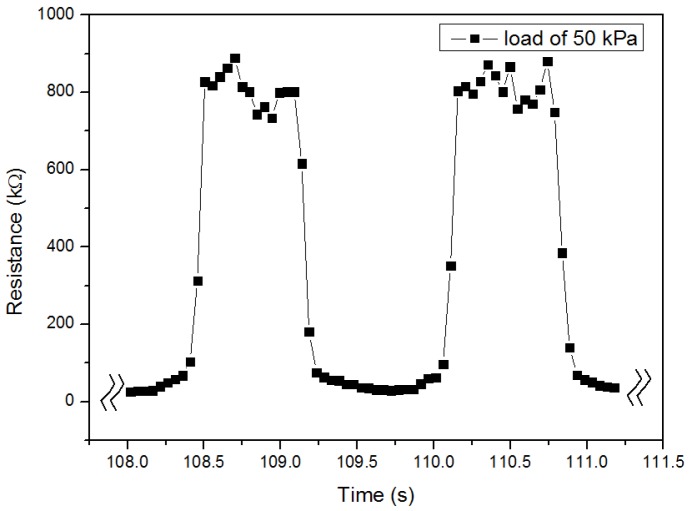
Response and recovery time of the pressure sensor. Concentration of TS029 in PDMS: 50 wt %.

**Figure 10:**
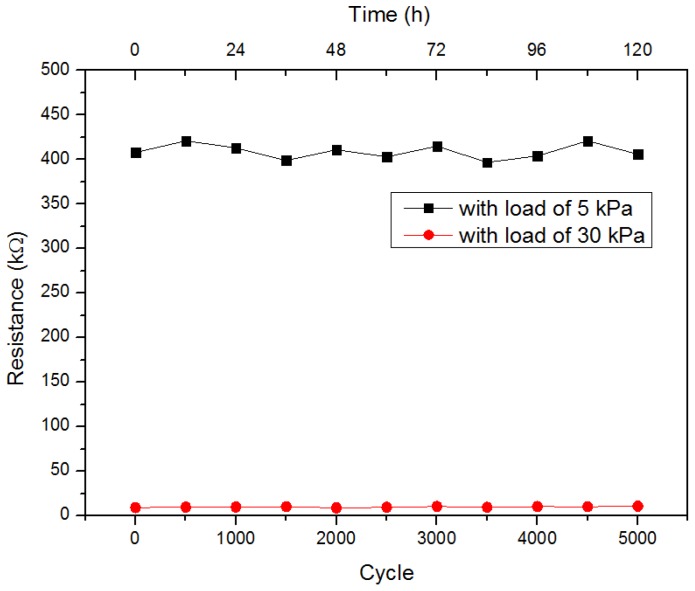
The stability and durability of the pressure sensor with TS029 concentration in PDMS of 50 wt %. The test was performed by applying and removing loads of 5 kPa and 30 kPa for 5000 cycles (500 cycles per 12 h), respectively.

As the pressure sensor with concentration of TS029 in PDMS of 50 wt % showed a high sensitivity, the response time and stability of the sensor was measured. When a pressure of 50 kPa was applied and removed on the sensor, the response time and recovery time were 0.097 s and 0.145 s, respectively, as shown in Figure 9. The stability and durability test of the sensor was carried out by applying and removing loads of 5 and 30 kPa for 5000 cycles (500 cycles per 12 h) respectively, as illustrated in Figure 10. The relative resistance change of the pressure sensor was 6.6% and 2.2% under 5 kPa and 30 kPa respectively, implying high stability and durability.

## 4. Conclusions

In conclusion, we propose a facile method to fabricate a highly sensitive resistive pressure sensor with a composite film comprising of MWCNT nanoparticles, liquid crystal, and PDMS elastomer. LC droplets embedded in PDMS elastomer decrease the Young’s modulus of the active layer and contribute to the construction of conductive paths. By optimizing the concentration of TS029 in PDMS elastomer, the resistive pressure sensor shows a high sensitivity of 5.35 kPa^−1^, fast response (<150 ms), high durability, and simple fabrication process. The pressure sensor offers great potential for applications in emerging wearable devices and electronic skins.

## Figures and Tables

**Table 1:** The molecular formula of TS029 mixture.

Molecular Formula	End-Groups
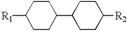	R_1_ = C_4_H_9_, R_2_ = C_3_H_7_
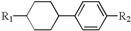	R_1_ = C_3_H_7_, R_2_ = C_2_H_5_
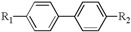	R_1_ = C_3_H_7_, R_2_ = CH_3_
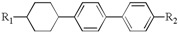	R_1_ = C_2_H_5_, R_2_ = C_3_H_7_
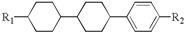	R_1_ = C_3_H_7_, R_2_ = CH_3_
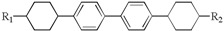	R_1_ = C_5_H_11_, R_2_ = C_3_H_7_
